# Accurate mitochondrial DNA sequencing using off-target reads provides a single test to identify pathogenic point mutations

**DOI:** 10.1038/gim.2014.66

**Published:** 2014-06-05

**Authors:** Helen R. Griffin, Angela Pyle, Emma L. Blakely, Charlotte L. Alston, Jennifer Duff, Gavin Hudson, Rita Horvath, Ian J. Wilson, Mauro Santibanez-Koref, Robert W. Taylor, Patrick F. Chinnery

**Affiliations:** 1 Wellcome Trust Centre for Mitochondrial Research, Institute of Genetic Medicine, Newcastle University, Newcastle-upon-Tyne, UK; 2 Wellcome Trust Centre for Mitochondrial Research, Institute for Ageing and Health, Newcastle University, Newcastle-upon-Tyne, UK

**Keywords:** exome, mitochondrial disorders, mitochondrial DNA, point mutation, sequencing

## Abstract

**Purpose:**

Mitochondrial disorders are a common cause of inherited metabolic disease and can be due to mutations affecting mitochondrial DNA or nuclear DNA. The current diagnostic approach involves the targeted resequencing of mitochondrial DNA and candidate nuclear genes, usually proceeds step by step, and is time consuming and costly. Recent evidence suggests that variations in mitochondrial DNA sequence can be obtained from whole-exome sequence data, raising the possibility of a comprehensive single diagnostic test to detect pathogenic point mutations.

**Methods:**

We compared the mitochondrial DNA sequence derived from off-target exome reads with conventional mitochondrial DNA Sanger sequencing in 46 subjects.

**Results:**

Mitochondrial DNA sequences can be reliably obtained using three different whole-exome sequence capture kits. Coverage correlates with the relative amount of mitochondrial DNA in the original genomic DNA sample, heteroplasmy levels can be determined using variant and total read depths, and—providing there is a minimum read depth of 20-fold—rare sequencing errors occur at a rate similar to that observed with conventional Sanger sequencing.

**Conclusion:**

This offers the prospect of using whole-exome sequence in a diagnostic setting to screen not only all protein coding nuclear genes but also all mitochondrial DNA genes for pathogenic mutations. Off-target mitochondrial DNA reads can also be used to assess quality control and maternal ancestry, inform on ethnic origin, and allow genetic disease association studies not previously anticipated with existing whole-exome data sets.

## Introduction

Mitochondrial disorders have emerged as a common cause of inherited metabolic diseases. The underlying biochemical defect affects the respiratory chain, which is encoded by genes within mitochondrial DNA (mtDNA) and the nuclear genome. Additional nuclear-encoded mitochondrial proteins are involved in mitochondrial biogenesis, the assembly of the respiratory chain, and the maintenance of mtDNA. As a result, mutations in both nuclear DNA and mtDNA can cause mitochondrial diseases.

Mitochondrial disorders characteristically cause an overlapping spectrum of disease. With a few notable exceptions, it is very difficult to predict the genetic defect responsible in a particular individual. Having excluded a handful of common mutations, the molecular diagnostic approach is guided by the muscle histochemistry or biochemistry in an affected tissue.^1^ This usually involves a series of investigations aimed at excluding large-scale deletions and depletion of mtDNA, followed by targeted resequencing of the mitochondrial and nuclear genomes.^2^ Even with recent diagnostic advances, this can be time consuming and expensive, and only yields a molecular diagnosis in approximately two out of three patients.^3^ The expanding clinical spectrum means that mitochondrial disorders enter the differential diagnosis of an increasing number of patients. There is therefore a clear need to develop a more streamlined and high-yield molecular approach.

Given its small size at ~16.5 kb, the majority of laboratories use a conventional Sanger sequencing of between 20 and 40 overlapping polymerase chain reaction (PCR) fragments from each patient. Although potentially less laborious, the targeted microarrays^4^ remain expensive, often require extensive crossvalidation with Sanger sequencing, and have limited capacity to detect novel insertion–deletion mutations. Bespoke next-generation sequencing panels provide an alternative,^5,6,7,8,9,10,11^ but given the growing number of nuclear genes implicated in mitochondrial diseases, comprehensive investigation ultimately leads to whole-exome or -genome sequencing. It is therefore of great interest that mtDNA sequences have been resolved through off-capture sequencing using conventional whole-exome sequence (WES) methods.^12,13,14^ Although there have been some notable successes,^15^ the reliability of this approach has not been evaluated, particularly in determining mtDNA heteroplasmy. Here we compare diagnostic Sanger sequencing and three “off-the-shelf” exome capture kits. We show that, providing there is a basic minimum base coverage, whole-exome capture will reliably detect mtDNA sequence variations with a low error rate similar to that of conventional Sanger sequencing, as well as detecting mtDNA heteroplasmies.

## Materials and Methods

We studied 46 patients with suspected mitochondrial disease. All patients underwent a diagnostic workup for mtDNA disease, including long-range PCR to detect mtDNA deletions, and Sanger mtDNA sequencing. Having excluded a pathogenic mtDNA mutation, we subjected the samples to WES to investigate a suspected Mendelian mitochondrial disorder, using the same genomic DNA sample. No insertion–deletion mutations were detected by Sanger sequencing, so we restricted our analysis of off-target WES reads to single-nucleotide variants (SNVs).

### WES and bioinformatic pipeline

The majority, 25 of 46, of patient DNA samples used for WES were extracted from whole blood, 8 were from muscle, and 13 were from skin fibroblasts. All DNA samples were extracted using the same standard procedure. Paired-end WES was performed using three different capture kits: SureSelect Human All Exon 50Mb Kit (Agilent, Berkshire, UK), TruSeq Exome Enrichment Kit 62Mb (Illumina, San Diego, CA), or SeqCap EZ Exome Library v2.0 (Roche Nimblegen, Madison, WI). See **Supplementary Table S1** online for sample-specific details. Sequences were aligned to the human reference sequence (UCSC hg19) (http://genome.ucsc.edu/) using either BWA v0.6.2 (ref. 16) or NovoAlign v2.07.13 (http://www.novocraft.com), were formatted using Samtools v0.1.18 (ref. 17), and the duplicate reads were removed using Picard v1.75 (http://picard.sourceforge.net/). SNVs were called using Varscan v2.2.2 (ref. 18) with a minimum total coverage of 5 reads and a minimum variant read depth of 3. The mitochondrial variants were annotated using Annovar v.21-Feb-2013 (ref. 19) and its mtDNA-specific database files (hg19_MT_GRCh37). Minor allele frequencies of variants were obtained from an in-house group of 269 exomes and also from 15,487 full-length mitochondrial sequences in NCBI-GenBank (downloaded June 2012), which were aligned using BWA-SW^20^ and variants called using Varscan v2.2.2 (ref. 18). Custom Perl scripts were used to convert the hg19 mtDNA variant positions and alleles to correspond to those of the revised Cambridge reference sequence (GenBank: NC_012920.1),^21^ to calculate sequence read coverage depth and to combine the minor allele frequencies with the annotated variants. mtDNA haplogroups were generated using Haplogrep^22,23^ (http://haplogrep.uibk.ac.at/) and previously described methods.^24^

### Real-time PCR

Real-time PCR was performed on the original DNA samples used for WES to quantify mtDNA copy number. Assessment of mtDNA copy number was carried out using a relative, real-time PCR assay using a Bio-Rad iQ5 Optical System (Hercules, CA) as described previously.^25^

### Mitochondrial genome sequencing

Sanger sequencing of the entire mtDNA genome was performed as described previously.^26^ Amplicons were sequenced using the BigDye v3.1 kit and capillary electrophoresed on an ABI3130xl Genetic Analyzer (Life Technologies, Warrington, UK). Alignment and variant calling were performed using SeqScape software (v2.1.1; Applied Biosystems, Paisley, UK), comparing with the GenBank reference sequence for human mtDNA (NC_012920.1).

### Assessment of mutation load by quantitative pyrosequencing

The mtDNA mutation load for specific alleles was assessed using quantitative pyrosequencing. The Pyromark Assay Design Software v2.0 (Qiagen, Crawley, West Sussex, UK) was used to design locus-specific PCR and pyrosequencing primers (**Supplementary Table S2** online) for each variant, and pyrosequencing was performed on the Pyromark Q24 platform according to the manufacturer's protocol. Quantification of the heteroplasmy level of each variant was achieved using Pyromark Q24 software to directly compare the relevant peak heights of the wild-type and the mutant nucleotides at the relevant position, as described previously.^27^

### Statistical analysis

The correlation plot, Pearson's correlation coefficients, Poisson test statistics, and binomial exact 95% confidence intervals (CIs) were generated using the R statistical package (v2.15.1) (http://CRAN.R-project.org/doc/FAQ/R-FAQ.html).

## Results

### Detecting mtDNA single-nucleotide variation in off-target WES reads

**Figure 1:**
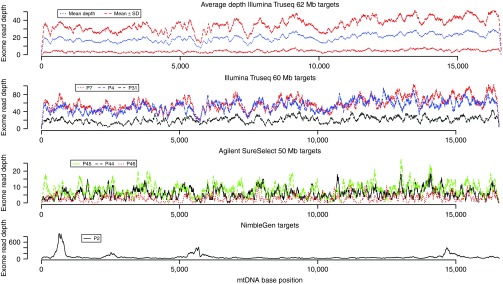
Average mitochondrial DNA (mtDNA) base read depth of 36 Illumina Truseq exomes and mtDNA base read depth for individual patient samples sequenced using three different whole-exome capture target kits.

An in-house WES analysis pipeline^28,29,30,31,32,33,34^ was used to detect SNVs in the mtDNA in genomic DNA samples from 46 patients. Exome sequences were generated using three different capture target kits (SureSelect Human All Exon 50Mb Kit (Agilent), TruSeq Exome Enrichment Kit 62Mb (Illumina), or SeqCap EZ Exome Library v2.0 (Nimblegen)) according to the manufacturers' protocols. Sequence reads were aligned to the human genome reference sequence (UCSC hg19) using either NovoAlign (http://www.novocraft.com) or BWA v0.6.2 (ref. 16) (see **Supplementary Table S1** online for details). The mean per-mtDNA-base read depth from the WES was 130.5-fold (minimum 2.5 and maximum 4,100.3), with just under two-thirds of the 46 samples exceeding a mean per-base coverage depth of 10-fold, and having greater than 90% of the mitochondrial genome covered to the minimum depth of 5-fold we required for variant calling (**Supplementary Figure S1** online). This large overall mean depth was due to three patients (P12, P13, and P32) with exceptionally high coverage (**Supplementary Table S1** online), the exclusion of which resulted in a more typical mean read depth of 25.7-fold (minimum 2.5 and maximum 206.6) for the remaining 43 patients. As shown before, the mtDNA sequences generated by the different capture target kits showed distinct patterns of per-base coverage depth,^14^ whereas sequences from the same capture targets largely followed the same coverage pattern but at varying read depths (**Figure 1**). A total of 402 different SNVs were detected by WES; 301 SNVs were intragenic, of which 245 were within coding genes; 63 variants were nonsynonymous; and 1 variant was stop-loss (**Supplementary Table S3** online). Eleven of the mtDNA variants were rare, with minor allele frequencies of less than 0.0001, and for which the minor allele frequencies were obtained from 15,487 full-length mtDNA sequences in GenBank and mtDNA variants from 269 in-house exome sequences.

### Off-target read depth correlates with the relative amount of mtDNA

Eight of the patients' exomes were generated from muscle DNA; seven of these exomes were generated using Illumina Truseq 62Mb capture targets and had greater than 99% of the mitochondrial genome covered to at least 5-fold, with mean per-base depths ranging from 28-fold to 4,100-fold (mean = 734-fold, *n* = 7). The eighth “muscle” exome was the only one from this group generated using Agilent 50Mb capture targets and had a mean per-mtDNA base depth of 2.5-fold with only 13.2% of bases covered to at least 5-fold. The coverage from the “muscle” group was higher than for exomes derived from both blood and fibroblast DNA, for which the mean coverage of mtDNA showed a range from 3-fold to 207-fold (blood exome mean = 22-fold, *n* = 25; fibroblast exome mean = 24-fold, *n* = 13). These findings were in keeping with previous observations,^14^ suggesting that off-target read depth is related to the relative number of mtDNA molecules in a given tissue sample.

**Figure 2:**
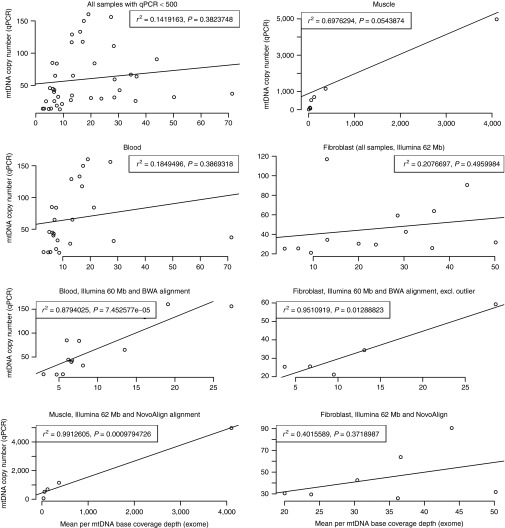
Correlation between mean mitochondrial DNA (mtDNA) per base depth from whole-exome sequence and mtDNA copy number from quantitative polymerase chain reaction (qPCR). qPCR measurements were performed in triplicate.

Real-time PCR was used to determine the mtDNA copy number for the 46 patients (data shown in **Supplementary Table S1** online). **Figure 2** shows the correlation between mtDNA copy number from quantitative PCR and mean mtDNA base read depth from WES; correlations for subgroups of tissue type, capture target, and WES data batch are also shown. The Pearson's correlation coefficient (*R*^2^) for 40 of the patients was 0.142 (*n* = 40, *P* = 0.382), in which 6 six patients were excluded from the analysis because their large values for mean coverage (>100-fold) and quantitative PCR (>500) were obscuring the results for the remaining samples. Restricting the analysis to the tissue subgroups increased the correlations, although they were not statistically significant (muscle: *R*^2^ = 0.698, *n* = 8, *P* = 0.054; blood: *R*^2^ = 0.185, *n* = 25, *P* = 0.387; fibroblast: *R*^2^ = 0.208, *n* = 13, *P* = 0.496). Significant correlations were found by further restricting the analysis to patients sequenced using the Illumina Truseq 62Mb capture targets and WES aligned with either BWA or NovoAlign (muscle/62Mb/NovoAlign: *R*^2^ = 0.9913, *n* = 5, *P* = 9.8 × 10^−4^; blood/62Mb/BWA: *R*^2^ = 0.880, *n* = 13, *P* = 7.5 × 10^−5^; fibroblast/62Mb/BWA: *R*^2^ = 0.951, *n* = 5, *P* = 0.013). However, nonsignificant correlations were found for the blood/fibroblast, Illumina Truseq 62Mb, and NovoAlign subgroups (blood/62Mb/NovoAlign: *R*^2^ = 0.160, *n* = 6, *P* = 0.762; fibroblast/62Mb/NovoAlign: *R*^2^ = 0.402, *n* = 7, *P* = 0.372). Similar correlations were obtained when the quantitative PCR data were compared directly with WES coverage in the region of the quantitative PCR amplicons. These observations confirm that, in general, off-target read depth appears to correlate with the relative amount of mtDNA, providing the DNA was extracted from the same tissue type and WES was generated by the same protocol and analysis pipeline.

### Validation of WES off-target mtDNA variants by the codetection of expected haplogroup markers

The haplogroups for each patient were predicted using all the SNV calls generated from WES for the 46 patients by using the Haplogrep software.^22,23^ The haplogroup predictions were all assigned quality scores of greater than 85%, with the exception of patient P1, who was predicted to belong to haplogroup R6a but with a lower-quality score of 73%. Patient haplogroup distribution (**Supplementary Table S4** online) was not significantly different from that of UK controls,^24^ suggesting that the SNV calls from WES appear to be genuine sequence variants and can be used to determine a patient's mitochondrial haplogroup. This may be of value in forensic and population genetic studies or as part of the quality-control procedure when analyzing WES data.

### Coverage threshold for the detection of WES off-target mtDNA variants

SNVs called by the analysis pipeline were compared with variants identified using diagnostic Sanger sequencing protocols. **Supplementary Table S5** online shows the number of SNVs detected in each patient for each sequencing technique relative to the revised Cambridge reference sequence (GenBank: NC_012920.1). A total of 762,174 bases of mtDNA were sequenced across the 46 patients. Sanger sequence detected SNVs at 1,277 bases, and WES detected SNVs at 1,110 bases; 1,079 SNVs were codetected by the two sequencing technologies. The vast majority of SNVs (185/198) that were detected only by Sanger sequencing corresponded to bases for which the WES read depth was less than the fivefold threshold for SNV calling. In total, 106,700 of 762,174 (14%) bases failed to meet the fivefold calling threshold, resulting in SNVs being missed by WES in 19 patients; only 6 patients had all 16,569 base pairs of their mtDNA completely “covered” (fivefold) by WES. This led us to propose a minimum mean per-mtDNA-base coverage threshold of 20-fold in WES to ensure that the vast majority of mtDNA SNVs are detected. In our data, 20 of 46 patients reached this threshold, with 330,684 of 331,380 (99.8%) bases sequenced to the minimum fivefold by WES and 0 of 512 SNVs missed due to low coverage.

**Tables 1** and **2** show 13 mtDNA SNVs were detected only by the original Sanger sequence, despite adequate WES read depth at each base (greater than fivefold), and 14 mtDNA SNVs were detected only by WES. Replicate Sanger sequencing was then performed from the original patient DNA across each one of these 27 variant sites. Furthermore, 10 of 13 SNVs originally detected by Sanger sequencing were not detected in the repeat Sanger sequence and were therefore likely to be sequencing artifacts from the initial Sanger sequencing. Of the remaining three SNVs, two (m.240A>G and m.16186C>T) could be seen in WES by manually evaluating the reads that showed potential heteroplasmy, but they had low variant base quality scores.

**Figure 3:**
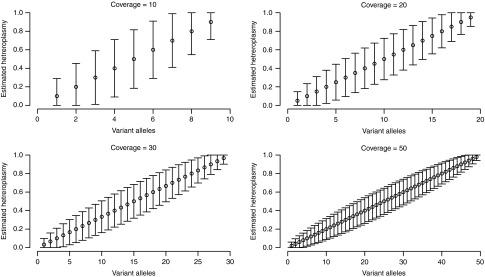
Binomial 95% confidence intervals for the detection of heteroplasmies at differing whole-exome sequence coverage depths. Intervals are shown for total read coverage depths (*n*) of 10-, 20-, 30-, and 50-fold, with the total range of variant allele counts (*v*) from 1 to *n* − 1, and the resulting estimated heteroplasmies (*v*/*n*) of between 0.0 and 1.0 (0–100%). The plots demonstrate a reduction in the size of the confidence intervals at increasing exome read depths, indicating that the higher the total depth, the closer the estimated heteroplasmy level is likely to be to the true heteroplasmy level.

Pyrosequencing from the original DNA sample identified these two variants as being 32 and 86% heteroplasmic as compared to heteroplasmy predictions from WES reads of 18% (95% CI: 4–43%, CIs based on the binomial distribution and allele read frequencies) and 17% (95% CI: 6–36%), respectively. For m.240A>G, the 95% CI for the WES heteroplasmy estimate (4–43%) included the pyrosequencing result (32%). The total WES read depth at m.240A>G was 17-fold, with 3 reads containing the variant allele (3 of 17 = 18%). Greater read coverage depth would decrease the width of these CIs and increase the accuracy of the WES heteroplasmy estimate, as demonstrated in **Figure 3**. On the other hand, the 95% CIs for the WES heteroplasmy estimate for m.16186C>T (6–36%) did not contain the value obtained by pyrosequencing (86%). m.16186C>T is located within a poly-C tract, which could have affected WES read alignment, thus explaining why the pyrosequencing heteroplasmy measurement was different from the WES estimate. The third Sanger-only variant (m.574A>C) was not identified by pyrosequencing; this variant is also located next to a poly-C tract, which may be interfering with the variant detection from both WES and pyrosequencing.

Overall, the false-positive rate for Sanger sequencing was 10 of 762,174 total or 0.0013%, which is in keeping with previous estimates and corresponds to the DNA polymerase fidelity.^35^ On the other hand, 7 of 14 SNVs (24 of 31 bases), which were only identified by WES off-target reads at >4-fold depth, were not seen in the repeat Sanger sequence. This corresponds to an error rate of 24 of 762,174 or 0.0031% for WES, which shows a marginally significant increase as compared to the Sanger rate (Poisson exact ratio test (*P* = 0.02431) of rate ratio = 1). However, considering that the false-positive calls from WES repeatedly occurred at the same bases located in the region of the poly-C sequence tract m.16184–16193, with only two WES false calls outside of this region, the false call rate from WES for the vast majority of the mitochondrial genome is actually significantly lower than that of Sanger sequencing (2 of 761,714 or 0.0003% (WES) vs. 10 of 761,714 or 0.0013% (Sanger); Poisson exact ratio test (*P* = 0.03857) of rate ratio = 1). Of the seven SNVs that were originally identified only by WES but subsequently confirmed by repeat Sanger sequence, five were located in the noncoding control region (m.250T>C, m.16093T>C, m.16150C>T, m.16343A>G, and m.16390G>A) and one was a synonymous SNV in *MTND2* (m.5009A>G), and so these were assumed to be of no functional consequence. The seventh SNV, which was located in *MTRNR2* (m.2905A>G) and appeared from the WES, reads to be 42% heteroplasmic (95% CI: 26–69%). Pyrosequencing estimated the *MTRNR2* variant to be 44% heteroplasmic, which fell within the confidence interval from the WES data.

### The detection of mtDNA heteroplasmy by off-target WES

Next we looked for evidence of heteroplasmic SNVs in 13 of the 46 patients who had a mean mtDNA read depth greater than 30-fold, focusing on heteroplasmy levels between 10 and 90%. All 13 of the patients had SNVs that appeared to be heteroplasmic, but all were at the same nine base positions and were therefore assumed to be false calls (**Supplementary Table S6** online). Three of the patients had SNVs that appeared to show heteroplasmy unique to the individual person (**Supplementary Table S7** online). Heteroplasmic variants were confirmed by pyrosequencing for three out of five of these variants, at levels that were within the 95% CIs for the estimates from WES reads. The other two variants were detected but were homoplasmic on the pyrosequencing. These false heteroplasmy predictions from the WES reads were assumed to have occurred due to erroneous “background” variant base calls of threefold and fivefold, as compared to most base positions, which had only a “background” variant call level of between zerofold and twofold.

## Discussion

Here we show that off-target WES reads can be used to reliably determine the mtDNA sequence, providing there is adequate read depth. With >20-fold mean coverage, the error rate is only marginally higher than that of conventional diagnostic Sanger sequencing, and if the WES errors that repeatedly occur at the poly-C sequence tract are taken into account, the error rate is marginally lower in WES as compared to Sanger sequencing. Given the potential role of mtDNA mutations in a broad range of human disease phenotypes, we suggest that mtDNA analysis should be included in a diagnostic exome analysis pipeline.

Despite the exciting prospect of acquiring this potentially revealing data “gratis,” any positive results should be interpreted with caution. Although only one sample was sequenced after Nimblegen capture, limiting our ability to make a formal comparison across all three capture platforms, the per-mtDNA-base coverage plots (**Figure 1**) generated from our study confirm previous findings describing four peaks with Nimblegen targets.^14^ These peaks correspond to nuclear-encoded mitochondrial sequences, leading to false mtDNA calls and potential heteroplasmy. On the other hand, here we show that nuclear-encoded mitochondrial sequences do not pose a problem for the accurate calling of mtDNA SNVs and detection of heteroplasmy when using Illumina Truseq (62 Mb) or Agilent SureSelect (50 Mb) targeted sequence and a standard WES analysis pipeline, in which there is no need for an additional realignment of mitochondrial sequence reads to the mitochondrial genome as has been previously suggested.^14^ However, with both of these platforms, we observed dips in coverage in the region of the origin of light-strand replication (Ori-L, **Figure 1**). Consistency between different capture technologies suggests that the reduced coverage reflects the local sequence context, making these regions vulnerable to sequencing error when the mean coverage is low. Furthermore, common pathogenic mtDNA mutations are found in approximately 1 out of 300 of the general population, so any sequencing result must be interpreted in the correct clinical context. It is also worth noting that rare, region-specific polymorphic variants may not be listed on public databases. Having a population-matched control database has proven invaluable in our experience, allowing the early identification and exclusion of likely polymorphisms.

Finally, although this study was not specifically designed to determine the threshold for detecting heteroplasmy, it was found that mtDNA heteroplasmies could be detected in our data at levels of between approximately 40 and 90% from WES when the sequence had a mean read coverage depth >30-fold. It remains to be seen whether WES data can be used to reliably detect lower levels of heteroplasmy, which is more likely to be possible at greater coverage depths, as shown in **Figure 3**. However, tissue-specific pathogenic mtDNA point mutations are a rare but well-recognized cause of mitochondrial disease, so the absence of a pathogenic mtDNA mutation in WES from blood DNA does not exclude the possibility of a pathogenic mtDNA point mutation in skeletal muscle. Given our finding that the WES of skeletal muscle DNA yields excellent mtDNA coverage, there is a strong argument in favor of WES of DNA extracted from a clinically affected tissue for comprehensive genetic analysis of mtDNA and relevant nuclear genes in a patient with suspected mitochondrial disease.

The reliable extraction of mtDNA sequence from WES data has several advantages. Geographically restricted mtDNA sequence motifs enable an evaluation of maternal ancestry and can thus be incorporated into quality-control algorithms when analyzing the nuclear DNA sequence. MtDNA genotyping may be of value for forensic or population genetic studies. Common mtDNA variants have been associated with several common diseases, and the acquired mtDNA sequence can be incorporated into the analysis of complex traits. Finally, given the broad clinical phenotype, mtDNA disorders enter the differential diagnosis of a wide range of diseases. Our analysis shows that WES data, aimed at the diagnosis of suspected nuclear genetic disorders, can also be used to diagnose pathogenic mtDNA mutations, providing the level of heteroplasmy is above the detection threshold. As a first-line test, currently WES would be a costly way of investigating a suspected mitochondrial disorder presenting with the classic phenotype of a specific mtDNA disease. However, as costs fall and the analysis becomes more automated, WES may become the first port of call for clinicians suspecting mitochondrial disease in the future.

## Disclosure

The authors declare no conflict of interest.

## Figures and Tables

**Table 1:**
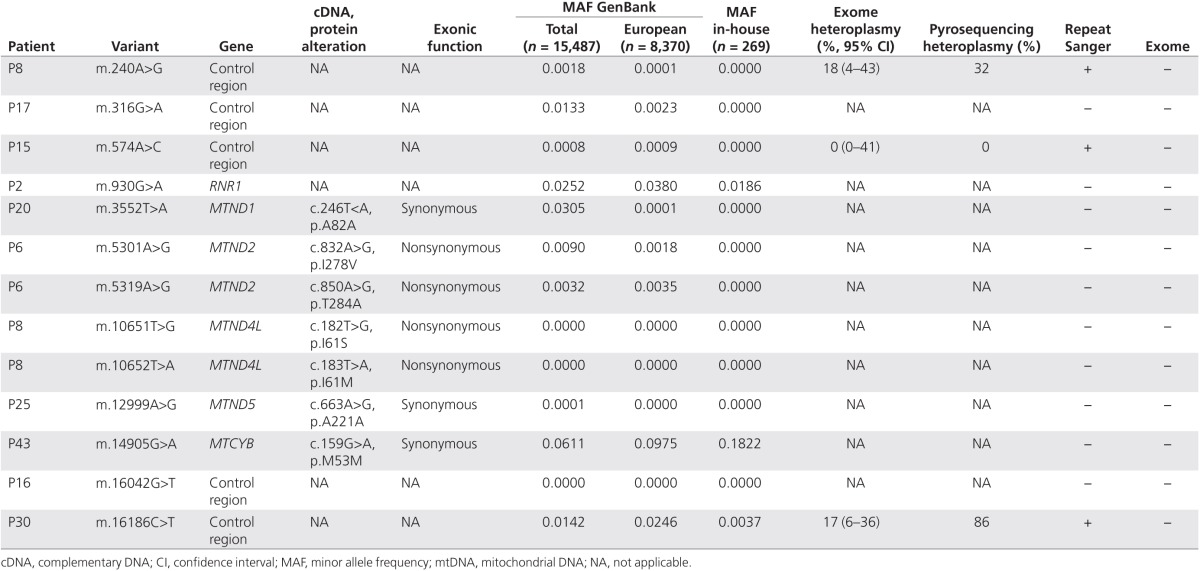
mtDNA variants detected only by Sanger sequencing (despite sufficient whole-exome sequence coverage)

**Table 2:**
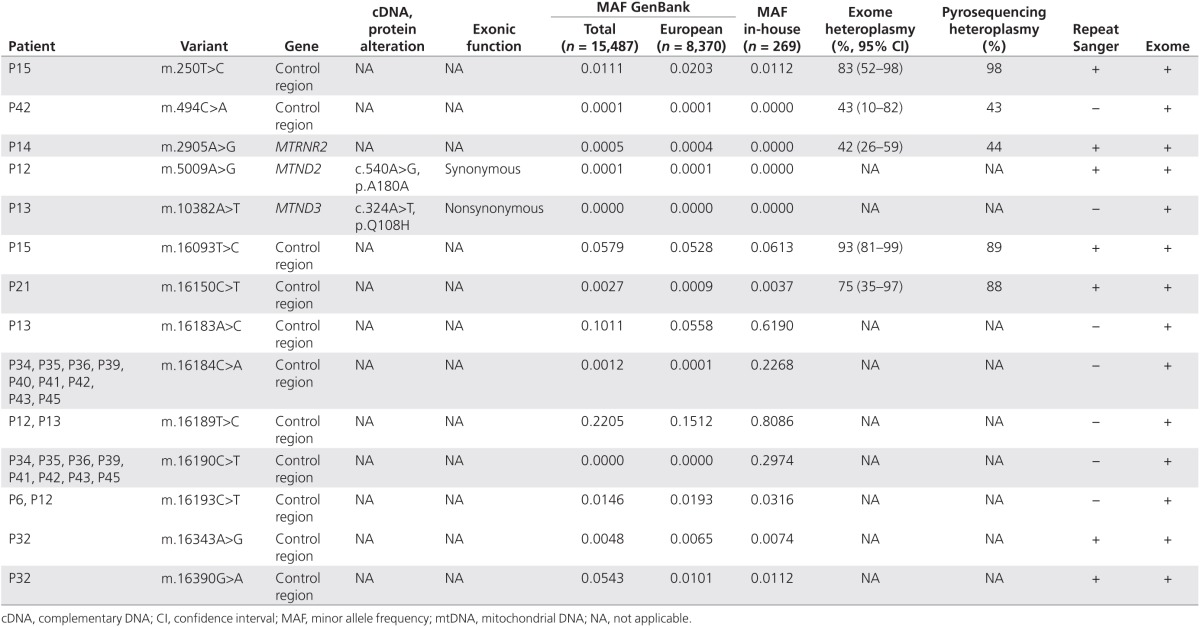
mtDNA variants detected only by whole-exome sequencing and not by Sanger sequencing
