# Supplementary material for: Accurate mitochondrial DNA sequencing using off-target reads provides a single test to identify pathogenic point mutations
Source: Genet Med. 2014 Jun 5;16(12):962–71. doi: 10.1038/gim.2014.66 (PMC4272251; doi:10.1038/gim.2014.66)
Supplement: Supplementary Figure S1 [file gim201466x1.doc]

**Supplementary Figure S1. Average per base coverage for mtDNA and percentage of mtDNA bases covered with a read depth of >5 fold derived from the whole exome sequence data in the 46 Patients.**

**
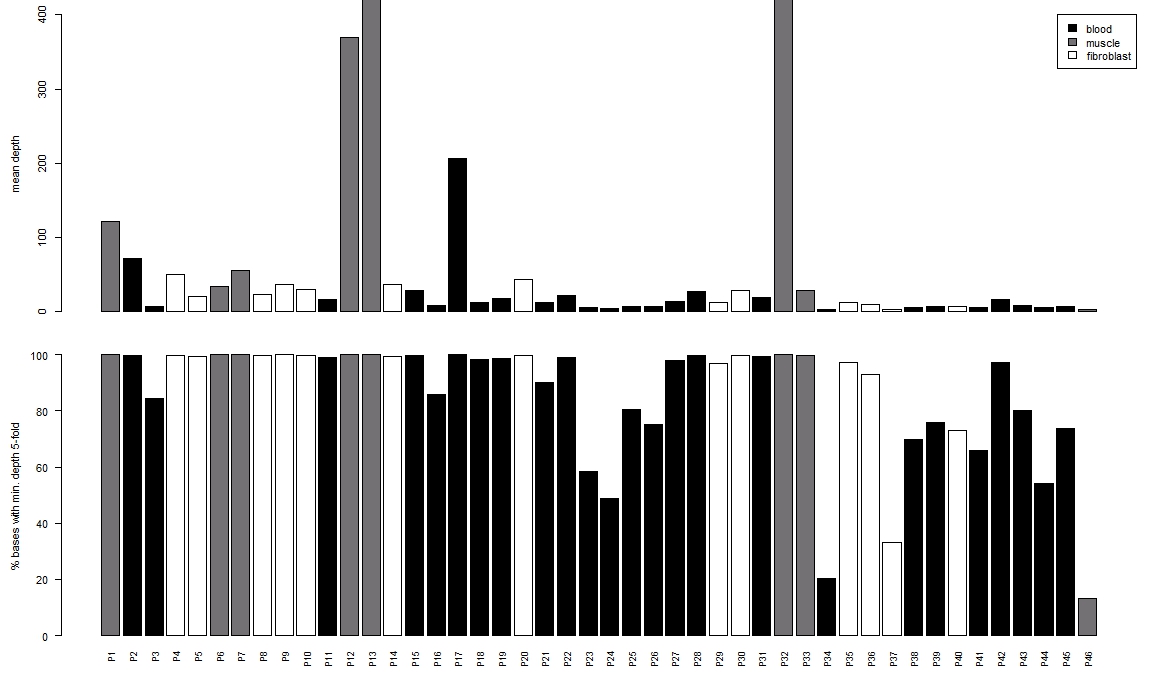
**
