# Supplementary material for: Accurate mitochondrial DNA sequencing using off-target reads provides a single test to identify pathogenic point mutations
Source: Genet Med. 2014 Jun 5;16(12):962–71. doi: 10.1038/gim.2014.66 (PMC4272251; doi:10.1038/gim.2014.66)
Supplement: Supplementary Table S1 [file gim201466x2.doc]

**Supplementary Table S1. Whole exome data and mtDNA levels determined by qPCR from the 46 patients**

| **ID** | **Company** | **Sequence Batch** | **Targets** | **Aligner** | **Tissue** | **Mean Target Base Depth** | **Min Depth** | **Max Depth** | **Number Bases 5-fold** | **% Bases 5-fold** | **Number Bases 1-fold** | **% Bases 1-fold** | **qPCR** | **Haplogroup** | **Haplogrep Quality (%)** |
| --- | --- | --- | --- | --- | --- | --- | --- | --- | --- | --- | --- | --- | --- | --- | --- |
| P1 | NIH | 1 | Illumina 62Mb | NovoAlign | Muscle | 121 | 9 | 184 | 16,569 | 100 | 16,569 | 100 | 694.6 | R6a | 73.2 |
| P2 | NIH | 1 | Nimblegen | NovoAlign | Blood | 71.3 | 0 | 913 | 16,538 | 99.8 | 16,568 | 100 | 37.1 | U5b2a4a | 97 |
| P3 | AROS | 2 | Illumina 62Mb | NovoAlign | Blood | 7.6 | 0 | 20 | 13,978 | 84.4 | 16,548 | 99.9 | 18.7 | H1+16239 | 100 |
| P4 | AROS | 2 | Illumina 62Mb | NovoAlign | Fibros | 50.2 | 1 | 93 | 16,557 | 99.9 | 16,569 | 100 | 31.8 | M3a1 | 92.8 |
| P5 | AROS | 2 | Illumina 62Mb | NovoAlign | Fibros | 20 | 0 | 39 | 16,456 | 99.3 | 16,559 | 99.9 | 30.47 | T2a1a | 91.4 |
| P6 | AROS | 3 | Illumina 62Mb | NovoAlign | Muscle | 34.5 | 2 | 67 | 16,567 | 100 | 16,569 | 100 | 66.78 | J1b1a1a | 98.3 |
| P7 | AROS | 3 | Illumina 62Mb | NovoAlign | Muscle | 55.4 | 3 | 100 | 16,569 | 100 | 16,569 | 100 | 522.98 | HV0+195 | 90.6 |
| P8 | AROS | 3 | Illumina 62Mb | NovoAlign | Fibros | 23.8 | 2 | 53 | 16,503 | 99.6 | 16,569 | 100 | 29.52 | H+73 | 85.2 |
| P9 | AROS | 3 | Illumina 62Mb | NovoAlign | Fibros | 36.2 | 3 | 78 | 16,564 | 100 | 16,569 | 100 | 25.97 | X2b+226 | 99.5 |
| P10 | AROS | 3 | Illumina 62Mb | NovoAlign | Fibros | 30.4 | 1 | 57 | 16,526 | 99.7 | 16,569 | 100 | 42.6 | U1a'c | 96.1 |
| P11 | AROS | 3 | Illumina 62Mb | NovoAlign | Blood | 17 | 0 | 36 | 16,395 | 98.9 | 16,561 | 100 | 117.64 | J1c3f | 93.8 |
| P12 | AROS | 3 | Illumina 62Mb | NovoAlign | Muscle | 369.4 | 20 | 579 | 16,569 | 100 | 16,569 | 100 | 1152.97 | K2a | 92.5 |
| P13 | AROS | 3 | Illumina 62Mb | NovoAlign | Muscle | 4100.3 | 217 | 7500 | 16,569 | 100 | 16,569 | 100 | 4976.8 | HV13 | 86.2 |
| P14 | AROS | 3 | Illumina 62Mb | NovoAlign | Fibros | 36.6 | 2 | 67 | 16,491 | 99.5 | 16,569 | 100 | 63.83 | H | 88.9 |
| P15 | AROS | 3 | Illumina 62Mb | NovoAlign | Blood | 28.5 | 1 | 59 | 16,543 | 99.8 | 16,569 | 100 | 31.4 | I4a | 93.2 |
| P16 | AROS | 3 | Illumina 62Mb | NovoAlign | Blood | 8.7 | 0 | 28 | 14,203 | 85.7 | 16,481 | 99.5 | 12.92 | H10e | 92.1 |
| P17 | AROS | 3 | Illumina 62Mb | NovoAlign | Blood | 206.6 | 7 | 446 | 16,569 | 100 | 16,569 | 100 | 1353.1 | U4a2 | 98.2 |
| P18 | AROS | 3 | Illumina 62Mb | NovoAlign | Blood | 13.1 | 1 | 28 | 16,297 | 98.4 | 16,569 | 100 | 129 | R30b1 | 90.6 |
| P19 | AROS | 3 | Illumina 62Mb | NovoAlign | Blood | 17.4 | 1 | 39 | 16,330 | 98.6 | 16,569 | 100 | 149.9 | T2b5 | 94.9 |
| P20 | AROS | 3 | Illumina 62Mb | NovoAlign | Fibros | 43.9 | 2 | 80 | 16,546 | 99.9 | 16,569 | 100 | 90.52 | M37+152+151 | 97.4 |
| P21 | OGT | 4 | Agilent 50Mb | NovoAlign | Blood | 12.7 | 1 | 216 | 14,951 | 90.2 | 16,569 | 100 | 27.2 | U1a3 | 94.8 |
| P22 | OGT | 4 | Agilent 50Mb | NovoAlign | Blood | 21.3 | 0 | 323 | 16,384 | 98.9 | 16,563 | 100 | 84.2 | H1c1 | 100 |
| P23 | AROS | 5 | Illumina 62Mb | BWA | Blood | 5.5 | 0 | 18 | 9,652 | 58.3 | 16,340 | 98.6 | 14.1 | U8a1a1a | 100 |
| P24 | AROS | 5 | Illumina 62Mb | BWA | Blood | 4.7 | 0 | 17 | 8,065 | 48.7 | 16,112 | 97.2 | 13.5 | H | 92.6 |
| P25 | AROS | 5 | Illumina 62Mb | BWA | Blood | 7.6 | 0 | 19 | 13,350 | 80.6 | 16,537 | 99.8 | 83.9 | T2b+16362 | 94.6 |
| P26 | AROS | 5 | Illumina 62Mb | BWA | Blood | 6.6 | 0 | 20 | 12,454 | 75.2 | 16,453 | 99.3 | 40 | H14a | 86.8 |
| P27 | AROS | 5 | Illumina 62Mb | BWA | Blood | 13.5 | 0 | 33 | 16,211 | 97.8 | 16,554 | 99.9 | 65.1 | H6a1a3 | 97.6 |
| P28 | AROS | 5 | Illumina 62Mb | BWA | Blood | 27.3 | 0 | 60 | 16,517 | 99.7 | 16,568 | 100 | 156.1 | U9b1 | 96.1 |
| P29 | AROS | 5 | Illumina 62Mb | BWA | Fibros | 13 | 0 | 29 | 16,086 | 97.1 | 16,542 | 99.8 | 116.9 | U5b2a2b1 | 98.7 |
| P30 | AROS | 5 | Illumina 62Mb | BWA | Fibros | 28.6 | 2 | 56 | 16,554 | 99.9 | 16,569 | 100 | 59.3 | H1c3 | 93.6 |
| P31 | AROS | 5 | Illumina 62Mb | BWA | Blood | 19.1 | 0 | 38 | 16,477 | 99.4 | 16,558 | 99.9 | 160.3 | K1a3a1b | 98 |
| P32 | AROS | 5 | Illumina 62Mb | BWA | Muscle | 427.1 | 1 | 702 | 16,569 | 100 | 16,569 | 100 | 5163.8 | U3a1 | 91 |
| P33 | AROS | 5 | Illumina 62Mb | BWA | Muscle | 28.3 | 0 | 51 | 16,524 | 99.7 | 16,551 | 99.9 | 111.1 | H13a1a1c | 100 |
| P34 | AROS | 6 | Illumina 62Mb | BWA | Blood | 3 | 0 | 11 | 3,385 | 20.4 | 15,530 | 93.7 | 14 | J2a1a1a2 | 96.7 |
| P35 | AROS | 6 | Illumina 62Mb | BWA | Fibros | 13.1 | 1 | 31 | 16,097 | 97.2 | 16,569 | 100 | 34.4 | U4b1a1a1 | 100 |
| P36 | AROS | 6 | Illumina 62Mb | BWA | Fibros | 9.5 | 0 | 28 | 15,432 | 93.1 | 16,559 | 99.9 | 21.2 | C4a3b | 91.9 |
| P37 | AROS | 6 | Illumina 62Mb | BWA | Fibros | 3.7 | 0 | 11 | 5,464 | 33 | 16,032 | 96.8 | 25.4 | L5c | 91.3 |
| P38 | AROS | 6 | Illumina 62Mb | BWA | Blood | 6 | 0 | 16 | 11,563 | 69.8 | 16,348 | 98.7 | 84.9 | X2l | 97.6 |
| P39 | AROS | 6 | Illumina 62Mb | BWA | Blood | 6.7 | 0 | 18 | 12,568 | 75.9 | 16,558 | 99.9 | 42.87 | J1c2o | 95.7 |
| P40 | AROS | 6 | Illumina 62Mb | BWA | Fibros | 6.7 | 0 | 20 | 12,099 | 73 | 16,456 | 99.3 | 25.6 | X2 | 91.8 |
| P41 | AROS | 6 | Illumina 62Mb | BWA | Blood | 6.2 | 0 | 21 | 10,935 | 66 | 16,330 | 98.6 | 44.5 | H6a1a8 | 100 |
| P42 | AROS | 6 | Illumina 62Mb | BWA | Blood | 16.1 | 0 | 47 | 16,117 | 97.3 | 16,540 | 99.8 | 133.2 | K1c2 | 98.3 |
| P43 | AROS | 6 | Illumina 62Mb | BWA | Blood | 8.1 | 0 | 22 | 13,293 | 80.2 | 16,429 | 99.2 | 32.4 | H13a1a | 96.2 |
| P44 | InHouse | 7 | Agilent 50Mb | BWA | Blood | 5.1 | 0 | 18 | 8,958 | 54.1 | 16,191 | 97.7 | 45.8 | J2a1a1a2 | 92.6 |
| P45 | InHouse | 7 | Agilent 50Mb | BWA | Blood | 7 | 0 | 23 | 12,240 | 73.9 | 16,381 | 98.9 | 64.99 | H1 | 92 |
| P46 | InHouse | 7 | Agilent 50Mb | BWA | Muscle | 2.5 | 0 | 11 | 2,189 | 13.2 | 14,391 | 86.9 | 13.9 | U2b2 | 98.6 |
