# Supplementary material for: Accurate mitochondrial DNA sequencing using off-target reads provides a single test to identify pathogenic point mutations
Source: Genet Med. 2014 Jun 5;16(12):962–71. doi: 10.1038/gim.2014.66 (PMC4272251; doi:10.1038/gim.2014.66)
Supplement: Supplementary Table S2 [file gim201466x3.doc]

**Supplementary Table S2. Pyrosequencing Primers**

This table displays the details of the primer sequences user for mutation load analysis by pyrosequencing. Each of the mutations is described along with the Gene Name and ID. Gene sequences were determined and numbered according to the revised Cambridge Reference Sequence (rCRS), NC_012920.1. Forward and reverse primers were designed for mtDNA amplification prior to pyrosequencing analysis. One primer in each pyrosequencing primer pair had a 5’ biotinylation modification (Bio-5’). An additional, sequencing primer was required for pyrosequencing analysis, which was used to assess mutational load. Pyrosequencing assays were designed using PyroMark assay design 2.0 from Qiagen.

|  | **Forward Amplification primer** | **Reverse Amplification primer** | **Pyrosequencing primer** |
| --- | --- | --- | --- |
| **Mutation Gene Name (ID)** |  |  |  |
|  | **5’ position sequence** | **5’ position sequence** | **5’ position sequence** |
| 2905A>G RNR2 | 28603 Bio-5’ TTCACCAGTCAAAGCGAACTACTA 3’ | 2908 5’ GGGTAACTTGTTCCGTTGGTCA 3’ | 2906 5’ TGTTCCGTTGGTCAAG 3’ |
|
| 5840C>T tRNA-Tyr | 5815 Bio-5’ CACCTCGGAGCTGGTAAAAA 3’ | 5858 5’ CTGAGTGAAGCATTGGACTGTAA 3’ | 5841 5’ GTAAATCTAAAGACAGGGG 3’ |
|
| 11719G>A ND4 | 11601 5’ ACCTAAAATCGCTCATTGCATAC 3’ | 11748 Bio-5’ GAGTGCGTTCGTAGTTTGAGTTT 3’ | 11703 5’CATAATCGCCCACGG 3’ |
|
| 16093T>C control reg | 16062 Bio-5’ ATTGACTCACCCATCAACAACC 3’ | 16097 5’ TGGTGGCTGGCAGTAATGTA 3’ | 16094 5’ CTGGCAGTAATGTACGA 3’ |
|
| 16271T>C control reg | 16181 Bio-5’ AAACCCCCTCCCCATGCTT 3’ | 16408 5’ GCGGGATATTGATTTCACGGAG 3’ | 16272 5’ GGGTAGGTTTGTTGGTAT 3’ |
|
| 240A>G control reg | 169 Bio-5’ ACGTTCAATATTACAGGCGAACA 3’ | 317 5’ TTTAAGTGCTGTGGCCAGAAG 3’ | 243 5’ TGTGCAGACATTCAATT 3’ |
|
| 574A>G control reg | 521 5’ ACACCGCTGCTAACCCCATAC 3’ | 750 Bio-5’ CTGCGTGCTTGATGCTTGTT 3’ | 557 5’ CCCAAAGACACCCCC 3’ |
|
| 16186C>T control reg | 16161 5’ TAAAAACCCAATCCACATCAAAA 3’ | 16270 Bio-5’ TGGGTAGGTTTGTTGGTATCCTAG 3’ | 16169 5’ CAATCCACATCAAAACC 3’ |
|
| 250T>C control reg | 169 Bio-5’ ACGTTCAATATTACAGGCGAACA 3’ | 364 5’GGTTAGGCTGGTGTTAGGGTTCTT 3’ | 251 5’AGTGGCTGTGCAGAC 3’ |
|
| 494C>A control reg | 406 Bio-5’ TTTGGCGGTATGCACTTTT 3’ | 582 5’ TTGCTTTGAGGAGGTAAGCTACA 3’ | 497 5’ GGGTAGGATGGGCGG 3’ |
|
| 16150C>T control reg | 16101 Bio-5’ TTACTGCCAGCCACCATGAAT 3’ | 16343 5’ GGGACGAGAAGGGATTTGACTGT 3’ | 16151 5’ TGGGTTTTTATGTACTACAG 3’ |
|
