# Supplementary material for: Accurate mitochondrial DNA sequencing using off-target reads provides a single test to identify pathogenic point mutations
Source: Genet Med. 2014 Jun 5;16(12):962–71. doi: 10.1038/gim.2014.66 (PMC4272251; doi:10.1038/gim.2014.66)
Supplement: Supplementary Table S3 [file gim201466x4.doc]

**Supplementary Table S3. mtDNA single nucleotide variants detected by whole exome sequencing**

| **Variant Type** | **Number of Variants** |
| --- | --- |
| Total Variants | 402 |
| Intra-genic | 301 |
| *MTRNR1* | 10 |
| *MTRNR2* | 24 |
| mt-tRNA | 22 |
| Coding genes | 245 |
| Synonymous | 181 |
| Non-Synonymous | 63 |
| Stop-loss | 1 |
| Rare (MAF<0.0001) | 11 |
| Non-Synonymous | 2 |
| mt-tRNA | 1 |
| *MTRNR1* | 1 |
| *MTRNR2* | 1 |
