# Supplementary material for: Accurate mitochondrial DNA sequencing using off-target reads provides a single test to identify pathogenic point mutations
Source: Genet Med. 2014 Jun 5;16(12):962–71. doi: 10.1038/gim.2014.66 (PMC4272251; doi:10.1038/gim.2014.66)
Supplement: Supplementary Table S4 [file gim201466x5.doc]

**Supplementary Table S4. Frequencies of mtDNA haplogroups of the 46 patients** determined from the whole exome sequence data as compared to average frequencies from the Wellcome Trust Case-Control Consortium (WTCC)

| **Geographic Location** | **Haplogroup** | **Frequency** | **%** | **Subgroups** | **Expected % (WTCC)** |
| --- | --- | --- | --- | --- | --- |
| Europe | R | 2 | 4.3 | R6a, R30b1 | - |
|  | HV | 2 | 4.3 | HV0+195, HV13 | - |
|  | H | 13 | 28.3 | H, H, H+73, H1, H1c1, H1c3, H1+16239, H6a1a3, H6a1a8, H10e, H13a1a, H13a1a1c, H14a | 44 |
|  | V | 0 | 0 | - | 3 |
|  | J | 5 | 10.9 | J1b1a1a, J1c2o, J1c3f, J2a1a1a2, J2a1a1a2 | 12 |
|  | T | 3 | 6.5 | T2a1a, T2b5, T2b+16362 | 10 |
|  | U | 10 | 21.7 | U1a'c, U1a3, U2b2, U3a1, U4a2, U4b1a1a1, U5b2a2b1, U5b2a4a, U8a1a1a, U9b1 | 14 |
|  | K | 3 | 6.5 | K1a3a1b, K1c2, K2a | 9 |
|  | W | 0 | 0 | - | 3 |
|  | X | 3 | 6.5 | X2, X2b+226, X2l | 2 |
|  | I | 1 | 2.2 | I4a | 4 |
| Asia | M | 2 | 4.3 | M3a1, M37+152+151 | - |
|  | C | 1 | 2.2 | C4a3b | - |
| Africa | L | 1 | 2.2 | L5c | - |
