# Supplementary material for: Accurate mitochondrial DNA sequencing using off-target reads provides a single test to identify pathogenic point mutations
Source: Genet Med. 2014 Jun 5;16(12):962–71. doi: 10.1038/gim.2014.66 (PMC4272251; doi:10.1038/gim.2014.66)
Supplement: Supplementary Table S5 [file gim201466x6.doc]

**Supplementary Table S5. Number of single nucleotide variants detected in the whole exome data from the 46 patients**

| **ID** | **Number Bases 5-fold** | **Total Bases** | **Sanger SNVs (Total unconfirmed)** | **Sanger SNVs (Total confirmed)** | **Sanger Only (Total)** | **Sanger Only (WES coverage <5-fold)** | **Sanger Only (WES missed)** | **Sanger Only (WES missed + confirmed by 2nd PCR)** | **Sanger & WES** | **WES Variants (Total unconfirmed)** | **WES Variants (total confirmed)** | **WES Only** | **WES only (confirmed by 2nd PCR)** |
| --- | --- | --- | --- | --- | --- | --- | --- | --- | --- | --- | --- | --- | --- |
| P1 | 16,569 | 16,569 | 35 | 35 | 0 | 0 | 0 | 0 | 35 | 35 | 35 | 0 | 0 |
| P7 | 16,569 | 16,569 | 15 | 15 | 0 | 0 | 0 | 0 | 15 | 15 | 15 | 0 | 0 |
| P12 | 16,569 | 16,569 | 34 | 34 | 0 | 0 | 0 | 0 | 34 | 37 | 35 | 3 | 1 |
| P13 | 16,569 | 16,569 | 15 | 15 | 0 | 0 | 0 | 0 | 15 | 18 | 15 | 3 | 0 |
| P17 | 16,569 | 16,569 | 30 | 29 | 1 | 0 | 1 | 0 | 29 | 29 | 29 | 0 | 0 |
| P32 | 16,569 | 16,569 | 24 | 24 | 0 | 0 | 0 | 0 | 24 | 26 | 26 | 2 | 2 |
| P6 | 16,567 | 16,569 | 40 | 38 | 2 | 0 | 2 | 0 | 38 | 39 | 38 | 1 | 0 |
| P9 | 16,564 | 16,569 | 28 | 28 | 0 | 0 | 0 | 0 | 28 | 28 | 28 | 0 | 0 |
| P4 | 16,557 | 16,569 | 36 | 36 | 0 | 0 | 0 | 0 | 36 | 36 | 36 | 0 | 0 |
| P30 | 16,554 | 16,569 | 14 | 14 | 1 | 0 | 1 | 1 | 13 | 13 | 13 | 0 | 0 |
| P20 | 16,546 | 16,569 | 30 | 29 | 1 | 0 | 1 | 0 | 29 | 29 | 29 | 0 | 0 |
| P15 | 16,543 | 16,569 | 33 | 33 | 1 | 0 | 1 | 1 | 32 | 34 | 34 | 2 | 2 |
| P2 | 16,538 | 16,569 | 34 | 33 | 1 | 0 | 1 | 0 | 33 | 33 | 33 | 0 | 0 |
| P10 | 16,526 | 16,569 | 28 | 28 | 0 | 0 | 0 | 0 | 28 | 28 | 28 | 0 | 0 |
| P33 | 16,524 | 16,569 | 13 | 13 | 0 | 0 | 0 | 0 | 13 | 13 | 13 | 0 | 0 |
| P28 | 16,517 | 16,569 | 29 | 29 | 0 | 0 | 0 | 0 | 29 | 29 | 29 | 0 | 0 |
| P8 | 16,503 | 16,569 | 18 | 16 | 3 | 0 | 3 | 1 | 15 | 15 | 15 | 0 | 0 |
| P14 | 16,491 | 16,569 | 12 | 12 | 0 | 0 | 0 | 0 | 12 | 13 | 13 | 1 | 1 |
| P31 | 16,477 | 16,569 | 34 | 34 | 0 | 0 | 0 | 0 | 34 | 34 | 34 | 0 | 0 |
| P5 | 16,456 | 16,569 | 40 | 40 | 0 | 0 | 0 | 0 | 40 | 40 | 40 | 0 | 0 |
| P11 | 16,395 | 16,569 | 33 | 33 | 0 | 0 | 0 | 0 | 33 | 33 | 33 | 0 | 0 |
| P22 | 16,384 | 16,569 | 11 | 11 | 0 | 0 | 0 | 0 | 11 | 11 | 11 | 0 | 0 |
| P19 | 16,330 | 16,569 | 36 | 36 | 2 | 2 | 0 | 0 | 34 | 34 | 34 | 0 | 0 |
| P18 | 16,297 | 16,569 | 37 | 37 | 0 | 0 | 0 | 0 | 37 | 37 | 37 | 0 | 0 |
| P27 | 16,211 | 16,569 | 15 | 15 | 0 | 0 | 0 | 0 | 15 | 15 | 15 | 0 | 0 |
| P42 | 16,117 | 16,569 | 33 | 33 | 0 | 0 | 0 | 0 | 33 | 36 | 33 | 3 | 0 |
| P35 | 16,097 | 16,569 | 33 | 33 | 1 | 1 | 0 | 0 | 32 | 34 | 32 | 2 | 0 |
| P29 | 16,086 | 16,569 | 30 | 30 | 0 | 0 | 0 | 0 | 30 | 30 | 30 | 0 | 0 |
| P36 | 15,432 | 16,569 | 49 | 49 | 2 | 2 | 0 | 0 | 47 | 49 | 47 | 2 | 0 |
| P21 | 14,951 | 16,569 | 37 | 37 | 5 | 5 | 0 | 0 | 32 | 33 | 33 | 1 | 1 |
| P16 | 14,203 | 16,569 | 14 | 13 | 3 | 2 | 1 | 0 | 11 | 11 | 11 | 0 | 0 |
| P3 | 13,978 | 16,569 | 10 | 10 | 0 | 0 | 0 | 0 | 10 | 10 | 10 | 0 | 0 |
| P25 | 13,350 | 16,569 | 37 | 36 | 4 | 3 | 1 | 0 | 33 | 33 | 33 | 0 | 0 |
| P43 | 13,294 | 16,569 | 13 | 12 | 2 | 1 | 1 | 0 | 11 | 13 | 11 | 2 | 0 |
| P39 | 12,568 | 16,569 | 34 | 34 | 2 | 2 | 0 | 0 | 32 | 34 | 32 | 2 | 0 |
| P26 | 12,454 | 16,569 | 15 | 15 | 6 | 6 | 0 | 0 | 9 | 9 | 9 | 0 | 0 |
| P45 | 12,240 | 16,569 | 11 | 11 | 2 | 2 | 0 | 0 | 9 | 11 | 9 | 2 | 0 |
| P40 | 12,099 | 16,569 | 25 | 25 | 6 | 6 | 0 | 0 | 19 | 20 | 19 | 1 | 0 |
| P38 | 11,563 | 16,569 | 27 | 27 | 4 | 4 | 0 | 0 | 23 | 23 | 23 | 0 | 0 |
| P41 | 10,935 | 16,569 | 14 | 14 | 5 | 5 | 0 | 0 | 9 | 11 | 9 | 2 | 0 |
| P23 | 9,652 | 16,569 | 29 | 29 | 14 | 14 | 0 | 0 | 15 | 15 | 15 | 0 | 0 |
| P44 | 8,958 | 16,569 | 38 | 38 | 16 | 16 | 0 | 0 | 22 | 22 | 22 | 0 | 0 |
| P24 | 8,065 | 16,569 | 9 | 9 | 7 | 7 | 0 | 0 | 2 | 2 | 2 | 0 | 0 |
| P37 | 5,464 | 16,569 | 71 | 71 | 49 | 49 | 0 | 0 | 22 | 22 | 22 | 0 | 0 |
| P34 | 3,385 | 16,569 | 40 | 40 | 31 | 31 | 0 | 0 | 9 | 11 | 9 | 2 | 0 |
| P46 | 2,189 | 16,569 | 34 | 34 | 27 | 27 | 0 | 0 | 7 | 7 | 7 | 0 | 0 |
| **Total** | **655,474** | **762,174** | **1,277** | **1,267** | **198** | **185** | **13** | **3** | **1,079** | **1,110** | **1,086** | **31** | **7** |
