# Supplementary material for: Accurate mitochondrial DNA sequencing using off-target reads provides a single test to identify pathogenic point mutations
Source: Genet Med. 2014 Jun 5;16(12):962–71. doi: 10.1038/gim.2014.66 (PMC4272251; doi:10.1038/gim.2014.66)
Supplement: Supplementary Table S6 [file gim201466x7.doc]

Supplementary Table S6. Estimated heteroplasmy (%) derived from whole exome sequence data – recurrent calls in 13 patients with >30-fold mean read depth

| **Variant** | **Number of patients showing heteroplasmy (>0% <100%)** | **Number of patients showing heteroplasmy (10-90%)** |
| --- | --- | --- |
| m.310T>C | 8 | 2 |
| m.3447A>G | 13 | 4 |
| m.16182A>C | 5 | 1 |
| m.16183A>C | 9 | 1 |
| m.16184C>A | 7 | 2 |
| m.16189T>C | 12 | 6 |
| m.16190C>T | 10 | 5 |
| m.16192C>T | 10 | 5 |
| m.16193C>T | 13 | 11 |
