# Supplementary material for: Accurate mitochondrial DNA sequencing using off-target reads provides a single test to identify pathogenic point mutations
Source: Genet Med. 2014 Jun 5;16(12):962–71. doi: 10.1038/gim.2014.66 (PMC4272251; doi:10.1038/gim.2014.66)
Supplement: Supplementary Table S7 [file gim201466x8.doc]

**Supplementary Table S7. Estimated heteroplasmy (%) derived from whole exome sequence data – 3/13 patients (>30-fold mean read depth) with unique calls**

| **Patient** | **Variant** | **Gene** | **cDNA, Protein Alteration** | **Exonic Function** | **% Exome Heteroplasmy (95% CI)** | **% Pyro-sequencing Heteroplasmy** |
| --- | --- | --- | --- | --- | --- | --- |
| P14 | m.2905A>G | *MTRNR2* | NA | NA | 42 (26-59) | 44 |
| P14 | m.5840C>T | mt-*tRNA*-Tyr | NA | NA | 67 (30-93) | 100 |
| P6 | m.11719G>A | *MTND4* | c.960G>A, p.G320G | synonymous | 88 (74-96) | 100 |
| P14 | m.16093T>C | Control Region | NA | NA | 86 (73-94) | 83 |
| P20 | m.16271T>C | Control Region | NA | NA | 63 (49-75) | 71 |
